# Propionate metabolism in a human pathogenic fungus: proteomic and biochemical analyses

**DOI:** 10.1186/s43008-020-00029-9

**Published:** 2020-05-05

**Authors:** Luiz Paulo Araújo Santos, Leandro do Prado Assunção, Patrícia de Souza Lima, Gabriel Brum Tristão, Matthias Brock, Clayton Luiz Borges, Mirelle Garcia Silva-Bailão, Célia Maria de Almeida Soares, Alexandre Melo Bailão

**Affiliations:** 1 Laboratório de Biologia Molecular, Instituto de Ciências Biológicas, Universidade Federal de Goiás, Goiânia, Brazil; 2 Universidade Estadual de Goiás, Itapuranga, Brazil; 3 Fungal Biology and Genetics Group, University of Nottingham, Nottingham, UK

**Keywords:** *Paracoccidioides*, Methylcitrate cycle, Differential metabolism, Propionate kinase

## Abstract

Fungi of the complex *Paracoccidioides* spp. are thermodimorphic organisms that cause Paracoccidioidomycosis, one of the most prevalent mycoses in Latin America. These fungi present metabolic mechanisms that contribute to the fungal survival in host tissues. *Paracoccidioides lutzii* activates glycolysis and fermentation while inactivates aerobic metabolism in iron deprivation, a condition found during infection. In lungs *Paracoccidioides brasiliensis* face a glucose poor environment and relies on the beta-oxidation to support energy requirement. During mycelium to yeast transition *P. lutzii* cells up-regulate transcripts related to lipid metabolism and cell wall remodeling in order to cope with the host body temperature. *Paracoccidioides* spp. cells also induce transcripts/enzymes of the methylcitrate cycle (MCC), a pathway responsible for propionyl-CoA metabolism. Propionyl-CoA is a toxic compound formed during the degradation of odd-chain fatty acids, branched chain amino acids and cholesterol. Therefore, fungi require a functional MCC for full virulence and the ability to metabolize propionyl-CoA is related to the virulence traits in *Paracoccidioides* spp. On this way we sought to characterize the propionate metabolism in *Paracoccidioides* spp. The data collected showed that *P. lutzii* grows in propionate and activates the MCC by accumulating transcripts and proteins of methylcitrate synthase (MCS), methylcitrate dehydratase (MCD) and methylisocitrate lyase (MCL). Biochemical characterization of MCS showed that the enzyme is regulated by phosphorylation, an event not yet described. Proteomic analyses further indicate that *P. lutzii* yeast cells degrades lipids and amino acids to support the carbon requirement for propionate metabolism. The induction of a putative propionate kinase suggests that fungal cells use propionyl-phosphate as an intermediate in the production of toxic propionyl-CoA. Concluding, the metabolism of propionate in *P. lutzii* is under regulation at transcriptional and phosphorylation levels and that survival on this carbon source requires additional mechanisms other than activation of MCC.

## INTRODUCTION

Paracoccidioidomycosis (PCM) is a systemic and granulomatous mycosis, caused by thermodimorphic fungi of the *Paracoccidioides* genus, a complex of organisms geographically restricted to Latin America with high prevalence in Brazil, Colombia, Venezuela and Argentina (Brummer et al. 1993). PCM presents the highest mortality rate among the systemic mycoses in Brazil, causing 148–171 deaths per year (Prado et al. 2009) and affecting mainly farm workers who are exposed to fungal propagules during soil manipulation (Franco et al. 2000). Once in human tissues, where the temperature is above 36 °C, the hyphal fragments or conidia differentiate into the pathogenic yeast form that establishes the infection (San-Blas et al. 2002). During the infectious process *Paracoccidioides* spp. face a hostile environment where they are exposed to several host-imposed stresses including high temperatures, oxidative and nitrosative stresses, nutrient deprivation and low pH. Therefore, it is required that these fungi sense and adapt to those conditions to survive in host tissues (Gonzalez and Hernandez 2015; Polke et al. 2015).

Throughout the course of infection and colonization of several host sites, pathogenic fungi are confronted with one common challenge, which is nutrient acquisition and utilization. Nutrient limitation is a common strategy employed by the host to hamper fungal survival. Furthermore, each host niche encountered by a microorganism may provide a different type of carbon source (Brock 2009; Pereira et al. 2009). Therefore, the ability to acquire nutrients from the host and a metabolic flexibility are central pieces of fungal virulence arsenal. Molecular and biochemical methods have been applied to understand the metabolic status of *Paracoccidioides* spp. yeast cells in conditions mimicking infection. Differential gene expression studies have shown that *P. lutzii* yeast cells employ fermentative metabolism while mycelial cells use aerobic routes for energy production (Felipe et al. 2005), which was subsequently confirmed by proteomic studies (Rezende et al. 2011). In contrast, *P. brasiliensis* (PB18) utilizes a more aerobic metabolism in yeast cells compared to hyphae (Araujo et al. 2019). Further, during mycelium-to-yeast transition metabolic pathways change to support membrane and cell wall remodeling (Bastos et al. 2007). Transcriptomic analysis in a murine in vivo model revealed that during liver infection glycolysis combined with alcoholic fermentation (Bailao et al. 2006; Costa et al. 2007) are activated to counteract the low oxygen tension (Lima Pde et al. 2015) and iron starvation (Parente et al. 2011). The expression profile of yeast cells incubated with human blood or plasma revealed lipid utilization under both conditions, whereas gluconeogenesis was up-regulated only in blood (Bailao et al. 2006; Bailao et al. 2007). In a lung infection model proteomics and transcriptomics data revealed that *P. brasiliensis* also utilizes the beta-oxidation pathway for energy production (Lacerda Pigosso et al. 2017). Taken together, these observations strongly suggest that *Paracoccidioides* spp. present a niche- and species-dependent metabolic adaptation during host infection.

Investigations on other fungal pathogens revealed similar adaptation mechanisms with striking species-dependent differences on the importance of individual pathways for virulence. Exposure of *Candida albicans* to neutrophils or macrophages up-regulates amino acid biosynthetic genes and displays a shift from fermentative to non-fermentative metabolism (Fradin et al. 2005; Lorenz et al. 2004). Studies with GFP-fusion reporter strains confirmed that growth in tissues stimulated glycolysis, whereas the glyoxylate cycle and gluconeogenesis dominate when phagocytized by host cells (Barelle et al. 2006). This is in agreement with attenuated virulence of glyoxylate cycle mutants of *C. albicans* with a defect in the key enzyme isocitrate lyase (*icl*) and unable to utilize fatty acids as nutrient source (Lorenz and Fink 2001). However, other pathogenic fungi are not strictly dependent on ICL for successfully host infection. *Aspergillus fumigatus* strains deleted for ICL revealed no attenuation in virulence (Schobel et al. 2007). Furthermore, although ICL is strongly induced in *Cryptococcus neoformans* upon macrophage contact, the *icl* deleted mutant displayed virulence like wild type strain (Rude et al. 2002). On the other hand, glycolysis impairment resulted in attenuated virulence in a murine cryptococcosis inhalation model by showing decreased persistence in central nervous system, while displaying adequate persistence in lungs (Price et al. 2011). Thus, pathogenic fungi take use of different metabolic strategies to survive in host niches with specific nutrient compositions.

In general, it is likely that microbial pathogens get exposed to odd-chain or branched-chain fatty acids, branched-chain amino acids or cholesterol during the course of infection. Degradation of these compounds yields propionyl-CoA and its accumulation can be toxic for the cell. Propionyl-CoA toxicity relies mainly on its ability to competitively inhibit Coenzyme A dependent enzymatic reactions such as pyruvate dehydrogenase, ATP:citrate lyase or succinyl-CoA synthetase (Brock and Buckel 2004). To avoid this toxic propionyl-CoA accumulation, organisms developed metabolic pathways to transform propionyl-CoA into non-toxic and energy-containing metabolites. Several bacteria and higher eukaryotes use the methylmalonyl-CoA pathway to convert propionyl-CoA into succinyl-CoA. Propionyl-CoA is carboxylated to yield (*S*)-methylmalonyl-CoA, which is isomerized to (*R*)-enantiomer by methylmalonyl-CoA epimerase. This reaction is followed by a carbon chain rearrangement into succinyl-CoA catalyzed by the vitamin B12 dependent methylmalonyl-CoA mutase (Botella et al. 2009; Kaziro and Ochoa 1964; Maruyama and Kitamura 1985; Savvi et al. 2008). Fungi don’t seem to contain a functional methylmalonyl-CoA mutase and a modified version of the beta-oxidation pathway was described in *C. albicans* for metabolism of propionyl-CoA with 3-hydroxypropionate as key intermediate (Otzen et al. 2014). However, the main propionate metabolizing route in fungi is the methylcitrate cycle (MCC) that is also found in several bacterial species. This pathway is characterized by an α-oxidation of propionyl-CoA into pyruvate. Propionyl-CoA is condensed with oxaloacetate via the key enzyme methylcitrate synthase yielding methylcitrate. Methylcitrate is isomerized into methylisocitrate by employing a dehydration via methylcitrate dehydratase and rehydration by aconitase. Finally, a methylisocitrate lyase cleaves methylisocitrate in pyruvate and succinate. The latter is converted back into oxaloacetate by tricarboxylic acid cycle (TCA) enzymes, whereas pyruvate can be used either as energy source or as building block in anabolic pathways (Brock et al. 2002).

The ability to promote propionyl-CoA turnover has been associated with virulence in pathogenic microbes. The disruption of the gene encoding for hydroxypropionate dehydrogenase in *C. albicans* strongly attenuates virulence in a murine model of systemic candidiasis (Otzen et al. 2014). In the bacterium *Mycobacterium tuberculosis*, a functional methylcitrate cycle is essential for survival in macrophages (Munoz-Elias et al. 2006). Similarly, the impairment of MCC in *A. fumigatus* by disruption of the MCS encoding gene reduced survival of conidia in lungs and attenuated virulence in an invasive aspergillosis model (Ibrahim-Granet et al. 2008). These observations confirm that pathogenic microorganisms utilize host nutrients that lead to the production of propionyl-CoA. Therefore, specific pathways are required not only to avoid toxic effects mediated by propionyl-CoA, but also to use it as a carbon source.

Efforts have been made to characterize the metabolic mechanisms used by members of the *Paracoccidioides* complex and preliminary transcriptomic and proteomic studies on propionyl-CoA metabolism indicate an involvement of the MCC in virulence. Cells undergoing mycelium-to-yeast transition induce MCC genes at transcriptional and translational levels (Bastos et al. 2007; Rezende et al. 2011). The contact of yeast cells with human blood and plasma that mimics dissemination also increases transcripts encoding MCC enzymes (Bailao et al. 2006; Bailao et al. 2007). Additionally, the induction of MCC transcripts in *P. lutzii* cells recovered from mouse tissues suggests a role of this pathway in fungal survival in the host milieu (Bailao et al. 2006). Accordingly, the macrophage environment also requires an active propionyl-CoA turnover in fungal cells (Parente-Rocha et al. 2015). Proteomics and transcriptomic analysis showed that carbon starvation also triggers an accumulation of MCC enzymes in *P. brasiliensis*. (Lima et al. 2014). Altogether, these observations strongly suggest that the methylcitrate cycle composes a metabolic requirement for *Paracoccidiodes *spp. survival during infection and, consequently, might be a virulence determinant in this human pathogen. In this study, we characterized the molecular aspects of propionate metabolism in *Paracoccidioides* spp.

## METHODS

### Fungal maintenance and growth conditions

*Paracoccidioides lutzii* strain 01 (ATCC-MYA-826) and *P. brasiliensis* isolate 18 (ATCC 32069) were maintained on the solid culture medium Fava-Netto (Fava Netto 1955) at 36 °C for the yeast form and at 23 °C for the mycelium form during 5 and 15 days, respectively. Log phase cells were obtained by the cultivation in liquid Fava-Netto medium under agitation for 72 h at 36 °C for yeast, and 5 days at 22 °C for mycelium. Cells were then washed twice with phosphate buffered saline (PBS: 0.14 mM NaCl, 2.7 mM KCl, 1.8 mM KH_2_HPO_4_, 10 mM Na_2_HPO_4_, pH 7.3) and subjected to further analyses. For assays using different carbon sources the MMcM medium was used (Restrepo and Jimenez 1980). Solid medium was prepared by the addition of 1% agarose. MMcM was supplemented with glucose and/or sodium propionate to a final concentration between 5 mM and 50 mM. The cell viability was assessed by using trypan blue in a hemocytometer chamber. For spot dilution growth analyses, Fava-Netto pre-grown yeasts were adjusted to 1 × 10^8^ cells/mL and serially diluted (1:10 dilutions). From each dilution a total of 10 μL was spotted in MMcM medium with the specified carbon source and grown at 36 °C for 7 days. For enzymatic assays, equal wet weight of yeast and mycelium cells were inoculated in MMcM liquid medium to perform an accurate normalization of MCS activity.

### Data mining and bioinformatics analysis of methylcitrate cycle enzymes

*A. fumigatus* and *Aspergillus nidulans* methylcitrate synthase, methylcitrate dehydratase and methylisocitrate lyase amino acid sequences (Maerker et al. 2005; Muller et al. 2011) were used to search for orthologous proteins encoded in *Paracoccidioides* spp. genomes (www.broadinstitute.org). For the propionyl-CoA syntethase we used an *Aspergillus nidulans* sequence (Zhang et al. 2004), and for methylmalonyl-CoA pathway the respective enzyme sequences from *Caenorhabditis elegans* and *M. tuberculosis* were used. The sequences were obtained from GenBank and the search was performed using BLASTP algorithm (Altschul et al. 1997). The subcellular localization and mitochondrial import signal sequences were obtained by using the database WoLF PSORT (http://www.genscript.com/psort/wolf_psort.html) and the online tool MitoProt (https://ihg.gsf.de/ihg/mitoprot.html), respectively. The intron/exon identification and synteny analysis was performed using the information available at *Paracoccidioides* spp. genome database (https://fungidb.org/fungidb/).

### RNA extraction and quantitative real time PCR (qRT-PCR)

The *Paracoccidioides* sp. yeast and mycelium cells were grown in the presence of glucose and/or propionate at 10 mM for 24 and 48 h. RNA was extracted as described elsewhere (Tristao et al. 2014). Briefly, cells were lysed using mechanical cell rupture (Mini-Beadbeater; Biospec Products Inc., Bartlesville, OK) and total RNA was extracted using TRI Reagent (Sigma- Aldrich, St. Louis, MO, USA) according to the manufacturer’s instructions and used as template for in vitro reverse transcription using the High Capacity RNA-to-cDNA kit (Applied Biosystems, Foster City, CA, USA). The cDNAs were used for quantification of transcript levels by qRT-PCR using SYBR Green PCR Master Mix (Applied Biosystems) in a StepOnePlus Real-Time PCR System (Applied Biosystems). The transcript levels of the analyzed genes were normalized against the expression levels of alpha tubulin gene (accession number: XM_002796593). Data were expressed as a mean of three independent biological replicates. Relative expression levels were calculated using the standard curve method for relative quantification (Bookout et al. 2006). Standard curves were generated by pooling the cDNAs from all conditions used, which were further serially diluted. Student’s *t*-test was applied in the statistical analyses. Primers used in qRT-PCR are shown in Table S1.

### Production and purification of recombinant MCS

The sequence of the mature *Pb*MCS without mitochondrial import peptide was used for production of the recombinant enzyme in *Escherichia coli*. The coding sequence was amplified from cDNA of *P. lutzii* isolate *Pb*01 and cloned into a modified pET43 plasmid in frame with a His-Tag sequence (Hortschansky et al. 2007). The plasmid was transferred into *E. coli* BL21 (DE3) Rosetta 2 cells (Novagen/Merck), which were used for overproduction of the recombinant enzyme. Protein production was induced by cultivation in Overnight Express Instant TB medium at 30 °C*.* Cells were collected by centrifugation and resuspended in buffer A (50 mM Tris/HCl, 150 mM NaCl, 10% glycerol, pH 8.0), and disrupted by sonication. Lysates were clarified by centrifugation and loaded onto a nickel-Sepharose 6 Fast Flow gravity-flow column (GE Healthcare). After six washes with buffer A containing 30 mM imidazole the protein was eluted in buffer A containing 200 mM imidazole. The recombinant protein purity was assessed by SDS-PAGE. Fractions with purified enzyme were combined and desalted using centrifugal filter devices (Merck). Then, glycerol was added to purified MCS and samples were stored at − 20 °C. Protein concentrations were determined by using the Bradford protein assay kit from Bio-Rad and bovine serum albumin was employed as standard.

### Protein extraction and enzymatic activity

#### Obtention of cell extracts

Yeast and mycelium cells from cultures in glucose and/or propionate at 10 mM were subjected to protein extraction as described elsewhere (Rezende et al. 2011) with some modifications. Cells were harvested by centrifugation and subjected to disruption in a Mini-Beadbeater with glass beads and extraction buffer (50 mM Tris-HCl pH 8.0; 150 mM NaCl). The lysate was clarified by centrifugation and protein samples were quantified as described above. Protein extract dephosphorylation was performed as described elsewhere (Cruz et al. 2011).

#### MCS activity

The methylcitrate synthase activity and biochemical parameters of recombinant MCS were determined as described previously (Brock et al. 2000). Briefly, the reaction mixtures (1 ml) contained 50 mM Tris-HCl (pH 8.0), 1 mM DTNB, 1 mM oxaloacetate, 0.2 mM propionyl-CoA and the assays were started by adding the protein extracts (3 μg). The reaction was followed spectrophotometrically by measuring the change in A_412_ at 25 °C. One unit of enzyme activity was defined as the amount of enzyme producing 1 μmol min^− 1^ of CoASH under the assay conditions.

#### Formamidase activity

Formamidase (FMD) activity was measured by ammonia generation, as described (Borges et al. 2005). Samples of 50 μL with 5 μg of protein extract were added to 200 μl of formamide substrate solution at a final concentration of 100 mM in 100 mM phosphate buffer, pH 7.4, and 10 mM EDTA. The incubation was proceeded at 37 °C for 30 min. Subsequently, 400 μl of phenol-nitroprusside and 400 μl of alkaline hypochlorite were added to the reaction tube, and the reaction was incubated for 6 min at 50 °C. Absorbance was then read at 625 nm. The amount of ammonia released was determined from a standard curve. One unit (U) of FMD activity was defined as the amount of enzyme required to hydrolyze 1 μmol of formamide (corresponding to the formation of 1 μmol of ammonia) per minute per milligram of protein.

### Production of anti-r*Pb*MCS polyclonal antibodies and western blot assays

All animal work was conducted in agreement with the National Council of Animal Work (Conselho Nacional de Controle de Experimentação Animal-CONCEA). The recombinant *Pb*MCS was used to generate specific polyclonal serum in mice. A total of 150 μg r*Pb*MCS were inoculated per mice in three injections (50 μg each) with 15 days intervals. The mice were euthanized 15 days after the last injection in a CO_2_ chamber and the blood was collected. The serum with polyclonal antibodies was obtained by centrifugation of blood samples.

Western blot analyses were performed as described before (Parente et al. 2011). Briefly, protein extracts from propionate and glucose grown yeast cells were electrophoresed in SDS-PAGE gels or subjected to 2D gel electrophoresis (de Arruda Grossklaus et al. 2013). Then, the proteins were transferred to nitrocellulose membranes and blocked with 5% dry fat milk in PBS. The membranes were reacted with anti-r*Pb*MCS polyclonal antibodies (1:500 dilution) and then hybridized with anti-mouse IGG coupled with alkaline phosphatase (1:2000 dilution). The reaction was developed with chromogenic substrates 5-bromo-4-chloro-3-indolylphosphate (BCIP) and nitroblue tetrazolium (NBT).

### Label free quantitative UPLC-MS^E^ proteomics

Proteomic analysis was performed with protein extracts from three biological replicates of cells grown in 10 mM propionate and 10 mM glucose. Protein sample aliquots (150 μg) in 50 mM ammonium bicarbonate pH 8.5 were subjected to trypsin digestion as previously described (Bailao et al. 2014). The digested samples were spiked with an internal standard (rabbit phosphorylase B, Waters Corp, Milford, MA) to reach the final concentration of 150 fmol μL^− 1^ of the glycogen phosphorylase. The digested peptides were analysed in triplicate by NanoUPLC-MS^E^ (Waters Corporation, Manchester, UK) using a NanoACQUITY system coupled to a Synapt mass spectrometer. Raw data obtained were processed and searched against database using ProteinLynx Global Server (PLGS) version 2.4 (Waters Corp) with Expression^E^ algorithm (Geromanos et al. 2009; Lima et al. 2014; Prado et al. 2015). PLGS was loaded with a specific *Paracoccidioides* sp. database (http://www.broadinstitute.org). The database was randomized to assess the false-positive rate of identification (4%). One missed cleavage site was allowed. Carbamidomethyl was specified as fixed modification and phosphorylation of STY and oxidation of methionine were set as variable modifications. Protein quantitation was based on the observed intensity measurements compared to the intensity measurements of the internal standard. A protein detected in all replicates presenting a variance coefficient less than 10% in all injections was used to normalize protein levels to accurately compare protein abundances between control and treated samples.

## RESULTS

### *Paracoccidioides* spp. utilizes propionate as a carbon source

**Fig. 1:**
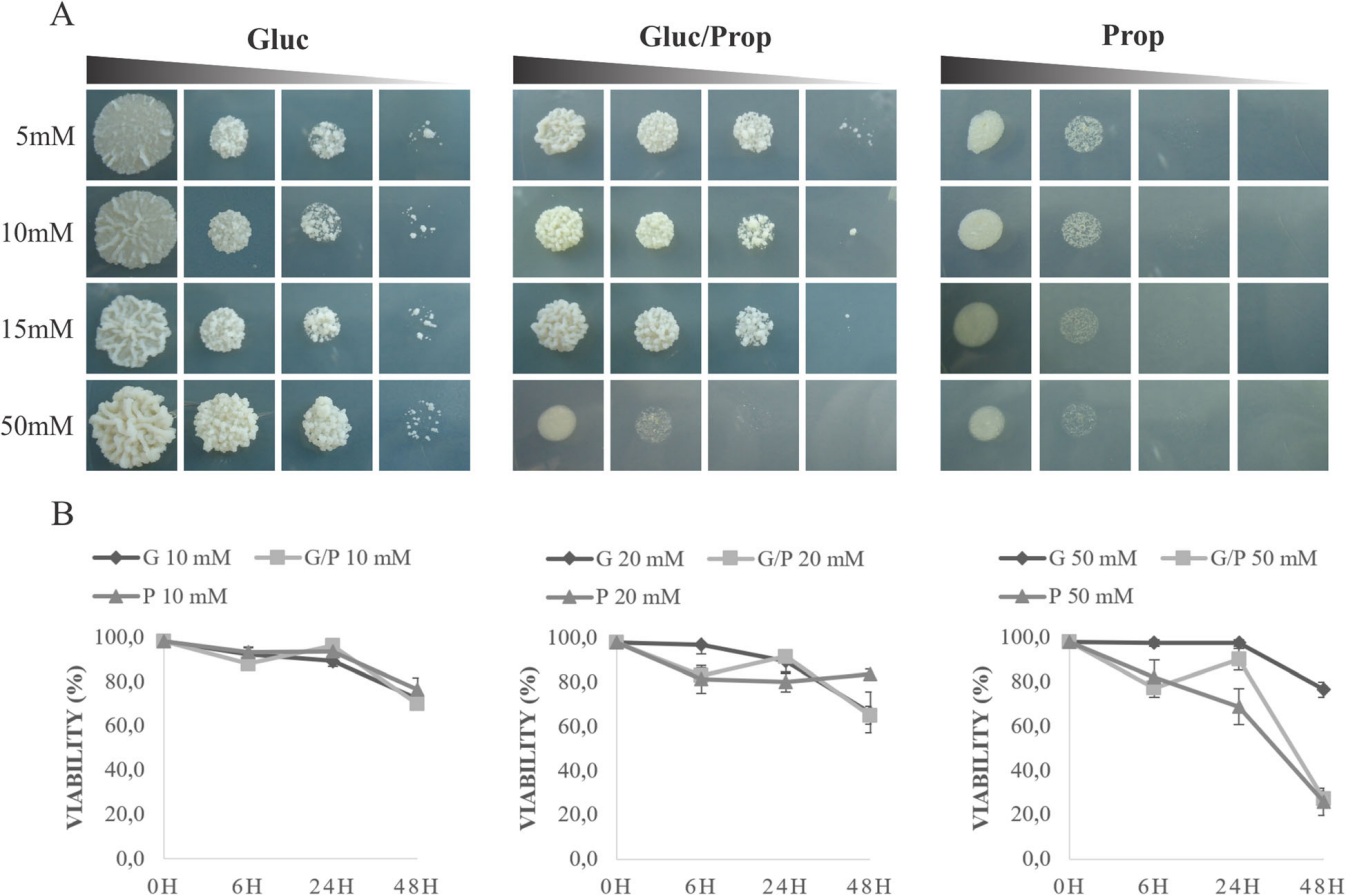
Growth and survival analysis of *P. lutzii* on propionate. **a** Serial dilutions of cell suspensions (from 10^6^ to 10^3^ cells) were spotted on MMcM containing propionate, glucose plus propionate and glucose as carbon sources. The plates were incubated at 36 °C for 10 days. **b** Viability analysis of yeast cells incubated on MMcM medium containing propionate and/or glucose from 10 to 50 mM. Cell viability was assessed by using trypan blue. Error bars represent the standard deviation from the average obtained from independent biological triplicates

*Paracoccidioides* spp. transcripts/enzymes related to propionate metabolism had been identified in infection-mimicking conditions (Bailao et al. 2006; Bailao et al. 2007; Lima et al. 2014; Parente et al. 2011; Pereira et al. 2009; Rezende et al. 2011). Therefore, fungal ability to grow and survive in the presence of propionate was analyzed. Initially, we monitored the growth in the presence of glucose, glucose/propionate and propionate in concentrations ranging from 5 mM to 50 mM (Fig. 1a). *Paracoccidioides* spp. growth in glucose/propionate was slightly affected at concentrations increasing to 15 mM of propionate and was strongly inhibited at 50 mM when compared to glucose. Propionate by itself supported fungal growth to a limited extent, since slow growth was observed at concentrations of 5 and 10 mM. Similar results were found using *P. brasiliensis* strain Pb18 (Figure S1). Additionally, since no difference in yeast cells growth were observed at 5 and 10 mM of propionate, we performed viability analysis at the concentrations 10 mM, 20 mM and 50 mM. Likewise, no difference in viability was detected among cells grown in glucose, glucose/propionate and propionate up to 24 h, although a slightly decrease occurred at 48 h in propionate (Fig. 1b). These results clearly demonstrate that glucose is the preferred carbon source when compared to propionate. Also, a high concentration of propionate is toxic for *P. lutzii* regardless the presence of glucose, as observed with other fungi (Brock et al. 2000; Maerker et al. 2005).

### Genomic characteristics of propionate metabolism in *Paracoccidioides* spp.

Microorganisms use variable routes or strategies to metabolize propionate in nature. The most common pathways used are the methylcitrate cycle and methylmalonyl-CoA pathway. Recently, it has been shown that *C. albicans* propionate degradation relies on a modified version of the beta-oxidation pathway (Otzen et al. 2014). To identify genes involved in propionyl-CoA degradation in the complex *Paracoccidioides*, we first screened the genomes for genes encoding enzymes of the methylcitrate cycle. For this analysis, the MCS, MCD and MCL of *A. nidulans* and *A. fumigatus* (Maerker et al. 2005; Muller et al. 2011) served as templates (Table S2). Our analysis revealed the presence of orthologs of all three- methylcitrate cycle specific enzymes in the three genomes analyzed (*P. lutzii*-Pb01, *P. americana*-Pb03 and *P. brasiliensis*-Pb18, Table 1). Also, the propionyl-CoA synthetase (PCS) from *A. nidulans* (CBF70755) (Zhang et al. 2004), an enzyme related to propionate activation by its conversion to propionyl-CoA, was used in BLASTP analysis. One homologues protein was identified in *Paracoccidioides* spp. genomes with identity of 74% and similarity of 84% (XP_002789592; Table 1; Table S2). The propionate degradation enzyme sequences presented high conservation among the *Paracoccidioides* species with identity ranging from 98 to 99%. Thus, the presence of genes coding to specific enzymes contributing to methylcitrate cycle suggest that the complex of *Paracoccidioides* spp. utilizes the MCC for degradation of propionate.

**Table 1:** Identity of *Paracoccidioides *spp. MCC enzymes when compared with functionally characterized enzymes

Enzymes	***An/Pl***	***An/Pb03***	***An/Pb18***	***Af/Pl***	***Af/Pb03***	***Af/Pb18***
PCL	74%	74%	74%	75%	74%	74%
MCS	78%	78%	78%	81%	81%	81%
MCD	78%	78%	79%	78%	78%	78%
MCL	77%	77%	74%	78%	78%	76%

*A. nidulans* (*An*), *A. fumigatus* (*Af*), *P. lutzii* (*Pl*), *P. americana* PS3 (*Pb03*) e *P. brasiliensis senso stricto* (*Pb18*)*. PCL* propionyl-CoA ligase, *MCS* methylcitrate synthase, *MCD* methylcitrate dehydratase, *MCL* methylisocitrate lyase

In order to identify genes required for a functional methylmalonyl-CoA pathway, we selected the essential enzyme methylmalonyl-CoA mutase for further genomics analysis. The analysis using the amino acid sequences of methylmalonyl-CoA mutases from *C. elegans* (accession number CAA84676) and *M. tuberculosis* (Rv1492 and Rv1493) (Savvi et al. 2008) shows no homologue in *Paracoccidioides* spp. Thus, it is unlikely that these fungi employ the methylmalonyl-CoA pathway in degradation of propionyl-CoA as previously shown for *A. nidulans* (Ledley et al. 1991).

**Fig. 2:**
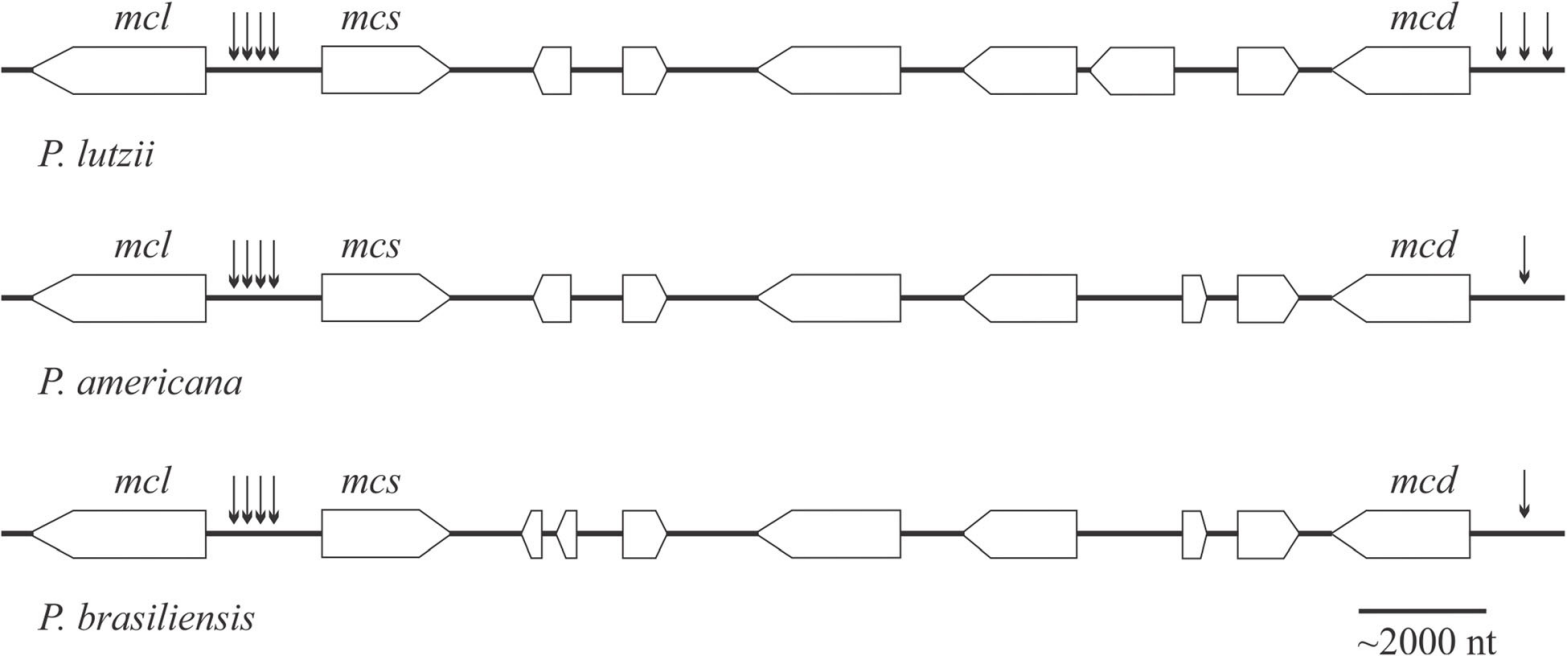
Genomic organization of methylcitrate cycle genes. Synteny of MCC genes in *Paracoccidioides *spp. genomes. The putative CreA binding motifs 5′-(G/C)YGGRG-3′ are denoted by arrows

Genes coding for enzymes related to a specific pathway or biological process are frequently clustered in the genome. Therefore, the synteny of MCC related genes was evaluated (Fig. 2). The three MCC specific genes are clustered in a region of approximately 22 kilobases. The *mcl* and *mcs* genes are located next to each other, while the MCD-encoding sequence is 16 kilobases away from MCS. Six (seven for *P. brasiliensis*) unrelated genes separate *mcd* from *mcs*. The catabolic repression phenomenon may play a role in the expression regulation of the MCC genes (Brock et al. 2000). CreA, a repressor acting when preferential carbon sources (such as glucose) are available, mediates this process in *A. nidulans* (Panozzo et al. 1998). The screening for CreA-binding sites in upstream regions of the genes revealed putative CreA binding sites (Fig. 2). Four binding sites were found in the promoter region shared by MCL and MCS in the three genomes; however, *P. americana* and *P. brasiliensis* presented two sites in each strand, while *P. lutzii* has three sites in positive strand and one in the negative strand. Using a 1000 bp region upstream MCD coding sequence, three CreA consensus sites were found in *P. lutzii* (two in positive strand and one in negative strand) and a single site was found in both *P. americana* and *P. brasiliensis*. These findings suggest that propionyl-CoA metabolism may be under control of a catabolic repressor system. Additionally, protein sequences of the MCC enzymes were analyzed for the presence of targeting signals by using the algorithm MitoProt. The results indicate a putative mitochondrial import signal for MCS, MCD and MCL whereas the putative PCS is predicted to be cytosolic; suggesting propionate metabolism likely occurs, at least in part, in mitochondrion, as reported for *Toxoplasma gondii (*Limenitakis et al. 2013*)*.

### Characterization of methylcitrate synthase

**Fig. 3:**
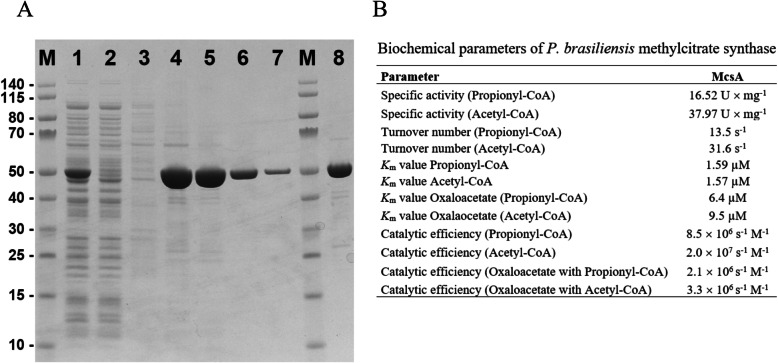
Production, purification and biochemical parameters of recombinant MCS. **a** -SDS-PAGE of different purification stages of MCS; Lane M – molecular weight marker, lane 1 whole bacterial cell lysate, lane 2 – flow-through from the Ni-resin, lane 3 – wash fraction through the resin, lanes 4–7 fractions eluted with 200 mM imidazole, lane 8 – concentrated pool of the MCS elution fraction (5 μg). **b** – Biochemical parameters of recombinant MCS

Although *Paracoccidioides* spp. present genes that likely encode methylcitrate synthase enzymes, the enzyme characteristics remained unknown for dimorphic fungi. Thus, MCS was produced as recombinant protein (r*Pb*MCS) for further analysis. To ease purification, the recombinant enzyme was fused to an *N*-terminal His-tag, whereby the predicted mitochondrial import sequence was omitted from the enzyme. After expression in *E. coli* the protein was purified by nickel-chelate chromatography. The purified fraction yielded a protein 92% pure as judged by SDS-PAGE analysis (Fig. 3a). Investigations of substrate specificity and specific activity revealed that propionyl-CoA serves as substrate with a specific activity of 16.52 U × mg^− 1^. Additionally, r*Pb*MCS showed significant activity as citrate synthase (using acetyl-CoA as substrate) with a specific activity of 37.97 U × mg^− 1^. In the presence of oxaloacetate the *K*_m_ for propionyl-CoA was determined as 6.4 μM while 9.5 μM for acetyl-CoA (Fig. 3b). It is important to highlight that the biochemical parameters are very similar to native methylcitrate synthase from *A. fumigatus* (Maerker et al. 2005) and *A. nidulans* (Brock et al. 2000), strongly suggesting this enzyme acts as a MCS in *P. lutzii*.

### Methylcitrate cycle enables *Paracoccidioides* spp. to metabolize propionate

**Fig. 4:**
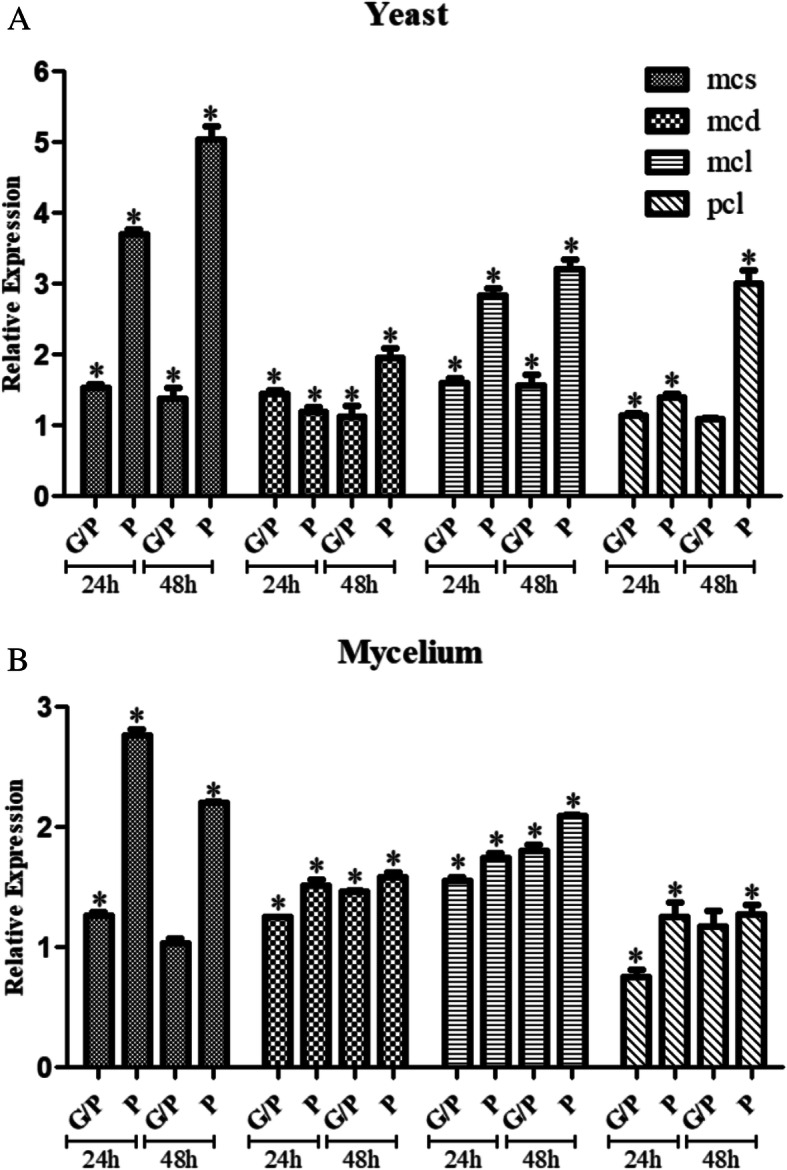
Expression analysis of the genes related to propionate metabolism on different carbon sources. Expression levels of the *mcs*, *mcd, mcl* and *pcl* genes from mycelium (**b**) and yeast (**a**) cells of *P. lutzii* grown in propionate (P) and glucose plus propionate (GP). The gene expression levels of propionate and glucose plus propionate were normalized to glucose condition (control carbon source). Error bars represent the standard deviation from the average obtained from triplicates; * *p* < 0.05

To determine the regulation of MCC cycle in *P. lutzii*, MCS, MCD, MCL and PCL transcript accumulation as well as MCS activity were measured in the presence of glucose and/or propionate. The qPCR analysis was performed in mycelium and yeast phases for MCS, MCD, MCL and PCS encoding genes. In general, data showed that propionate up-regulates all the genes even in presence of glucose for both forms (Fig. 4). However, the induction is significantly higher when propionate is the sole carbon source. MCS was the most regulated gene, in both mycelium and yeast, presenting induction rates of up to 5-fold, which corroborates with the fact that this enzyme catalyzes the first irreversible step of the MCC initiating propionyl-CoA detoxification (Domin et al. 2009). Predominantly, the up-regulation of MCC related genes, upon propionate addition, in mycelium was less intense when compared to yeast cells.

**Fig. 5:**
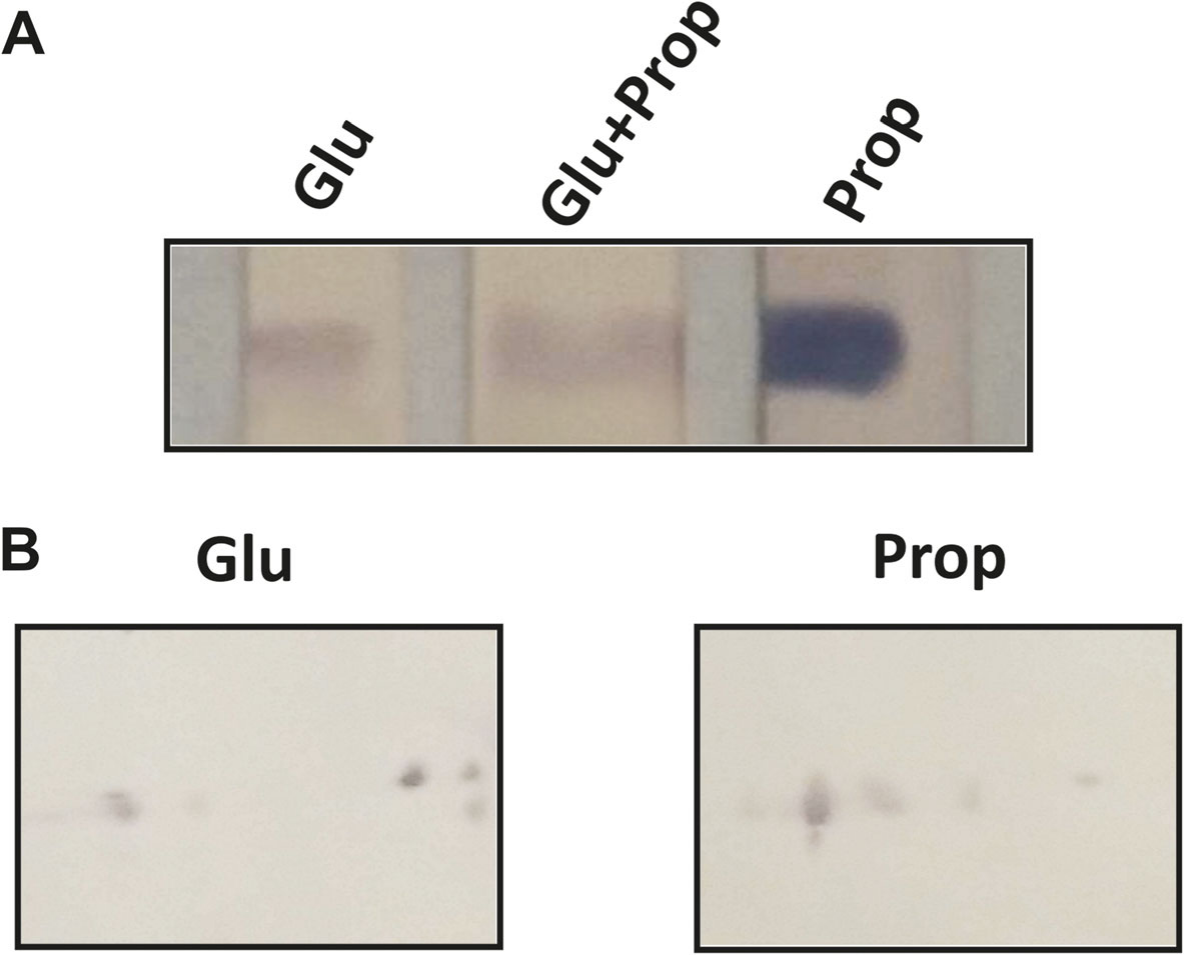
MCS levels and profiles in fungal cells grown in glucose or propionate. The protein extracts from yeast cells grown on propionate (Prop) and/or glucose (Glu) were subjected to 1-dimensional (**a**) or 2-dimensional (**b**) Western Blot analysis by using polyclonal antibodies raised against rPbMCS. Secondary anti-mouse antibodies coupled to alkaline phosphatase were used and the reaction was developed with BCIP and NBT

Western blot analysis using specific rPbMCS antibodies revealed an accumulation of the respective protein in yeast cells grown in propionate (Fig. 5). In order to confirm the presence of a functional MCS in vivo, enzyme activity was measured in cells grown in propionate or glucose. In both mycelium and yeast cells, MCS activity was induced in the presence of propionate (Table 2). The induction rate in yeast cells was higher than for mycelium corroborating the transcriptional data. Also, MCS activity in mycelium grown in glucose was higher than yeast cells in propionate. Intriguingly, *P. lutzii* maintained an elevated methylcitrate synthase activity even when glucose is the sole carbon source. Two-dimensional western blot data revealed a different pattern in isoform distribution with low mass variation, suggesting the presence of different phosphorylation patterns in the two samples (glucose and propionate, Fig. 5). In order to evaluate whether MCS is under a phosphorylation control mechanism, enzyme activity was measured in protein extracts subjected to phosphatase treatment. The dephosphorylation event promoted a two-fold reduction in MCS activity in both glucose and propionate conditions indicating that MCS is activated upon phosphorylation (Table 2). The MCS activity of dephosphorylated extracts from propionate grown cells were lower than in the non-dephosphorylated extracts obtained from glucose-grown cells.

**Table 2:** Methylcitrate synthase activity in cell extracts of yeast and mycelial forms of *P. lutzii* incubated in glucose, glucose with propionate or only propionate

	Condition	MCS (SD)	MCS (SD) Dephosphorylated
**Yeast**	Glucose	0.510 (±0.005)	0.224 (±0.002)
	Glucose/Propionate	0.708 (±0.005)	0.307 (±0.004)
	Propionate	0.952 (±0.009)	0.391 (±0.008)
**Mycelium**	Glucose	2.421 (±0.068)	–
	Glucose/Propionate	1.711 (±0.047)	–
	Propionate	1.961 (±0.081)	–

The data are defined as U × mg protein^− 1^. One unit of enzyme activity was defined as the amount of enzyme producing 1 μmol min^− 1^ of CoASH under the assay conditions. SD standard deviation; MCS methylcitrate synthase activity

### Metabolic strategies used by *Paracoccidioides* sp. during growth on propionate

Our results strongly suggest that *P. lutzii* is able to metabolize propionate and use it as carbon and energy sources. However, global analysis of the mechanisms involved in fungal adaptation to propionate remains unclear. Thus, we conducted a proteomic analysis of yeast cells grown in propionate in comparison to glucose. The quality analysis of the proteomic data showed 85 and 89% of the identified peptides were matched with 15 ppm error for glucose and propionate samples, respectively. Additionally, the peptide match distribution, which refers to the quality of the data at the peptide level, demonstrated that 11% (propionate sample) and 14% (glucose sample) of the peptides were in source and majority of the identified peptides was befitting with fragmentation in the trap CID cell. A total of 61 and 57% of the peptides was matched in the database without any modification in samples from glucose and propionate grown cells, respectively. Those numbers indicate the reliability of the proteomic data.

**Fig. 6:**
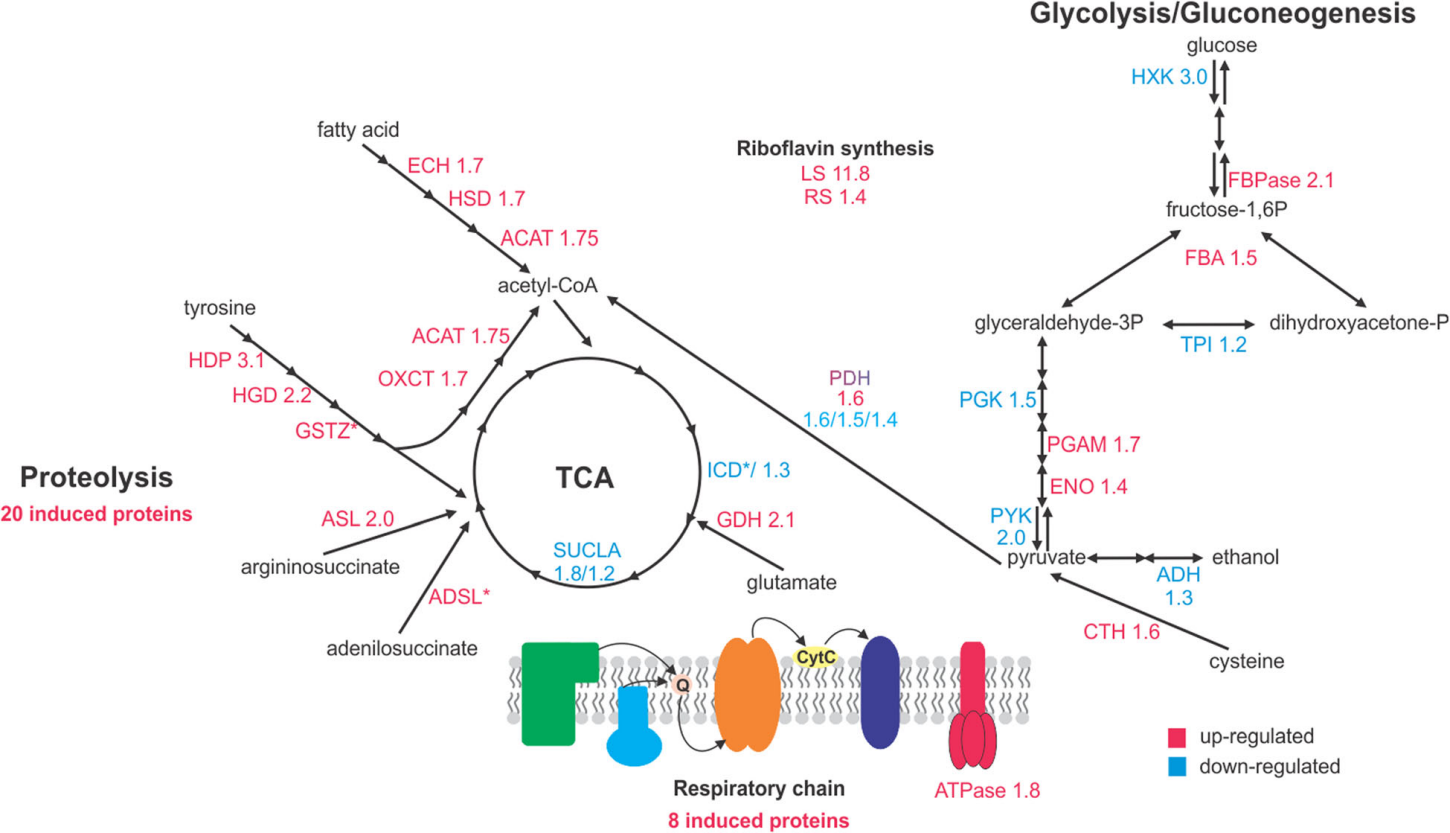
Metabolic changes of *P. lutzii* cells grown in propionate as deduced from proteomics. HXK hexokinase; PGI glucose-6P isomerase; ALD aldolase; TPI triose phosphate isomerase; PGAM phosphoglycerate mutase; PKM pyruvate kinase; ENO enolase; ADH alcohol dehydrogenase; PDH pyruvate dehydrogenase; HGD homogentisate 1,2-dioxygenase; HDP 4-hydroxyphenylpyruvate dioxygenase; MAIA maleyl acetoacetate isomerase; SCOT succinyl coenzyme A-acetoacetyl coenzyme A-transferase; ECH enoyl-CoA hydratase; ACAT acetyl-CoA acetyltransferase; ACO aconitase, IDH isocitrate dehydrogenase; KGD 2-oxoglutarate dehydrogenase; SDH succinate dehydrogenase; MDH malate dehydrogenase; FUM fumarate hydratase; ADSS adenylosuccinate synthetase; ADSL adenylosuccinate lyase; CTH cystathionine gamma lyase; MSY cobalamin independent methionine synthase; ARG arginase; CLY ATP-citrate lyase; FASa fatty acid synthase α; FASb fatty acid synthase β. The numbers indicate ratios of the induction (red) or repression (blue) of the proteins. * Enzymes detected only in glucose (blue) or propionate (red)

Comparison of the proteomic profiles obtained from propionate versus glucose utilizing yeasts identified 331 proteins with different amounts, of which 176 were induced in the presence of propionate (Tables S3 and S4). Functional categorization indicates that proteins related to general metabolism and energy production were the most influenced ones (Tables S3 and S4). The growth in propionate induced the degradation of fatty acid, with up-regulation of three β-oxidation enzymes. The accumulation of several enzymes related to amino acid degradation and protein degradation denotes that amino acids are required to support propionate metabolism. The final products of these processes might be fueling TCA and MCC to increase the propionyl-CoA turnover. The induction of enzymes aminotransferases, carbamoyl-phosphate synthase and glutamate dehydrogenase, related to nitrogen metabolism, would support adequate nitrogen metabolization. Thus, amino acid degradation would be providing TCA intermediates to support adaptation to this specific carbon source (Eoh and Rhee 2014). These observations are in agreement with previous work that observed a higher maintenance requirement necessary for growth in propionate (Brock and Buckel 2004). In propionate, the gluconeogenic enzyme FBPase-1 was up regulated while the glycolytic enzymes hexokinase and pyruvate kinase were down regulated. The induction of fatty acid oxidation, glyoxylate cycle (isocitrate lyase) and gluconeogenesis suggests fatty acids are being converted to glucose for structural purposes. Also, the joint induction of fatty acid and amino acid degradation pathways suggests the glucose-mediated catabolic repression mechanism is released since propionate is not a preferential carbon source. In a similar scenario (carbon starvation) *P. lutzii* induces the expression of the antigenic protein formamidase (FMD) (Borges et al. 2005; Lima et al. 2014). Growth in propionate also increases FMD levels in *P. lutzii* suggesting that this enzyme is required when glucose is absent. In order to confirm this theory, formamidase activity was measured. The enzyme activity in cells grown in propionate was 15 U × mg^− 1^, while in glucose cells the FMD activity was only 7.8 U × mg^− 1^. Intriguingly, riboflavin synthesis seems to be necessary for metabolic adaptation, since lumazine synthase (11.8 fold) and riboflavin synthase (1.4 fold) levels were increased. This couples with the induction of the riboflavin dependent enzymes succinate dehydrogenase and acyl-CoA dehydrogenase. An overview of the metabolic adaptation is summarized in Fig. 6.

**Fig. 7:**
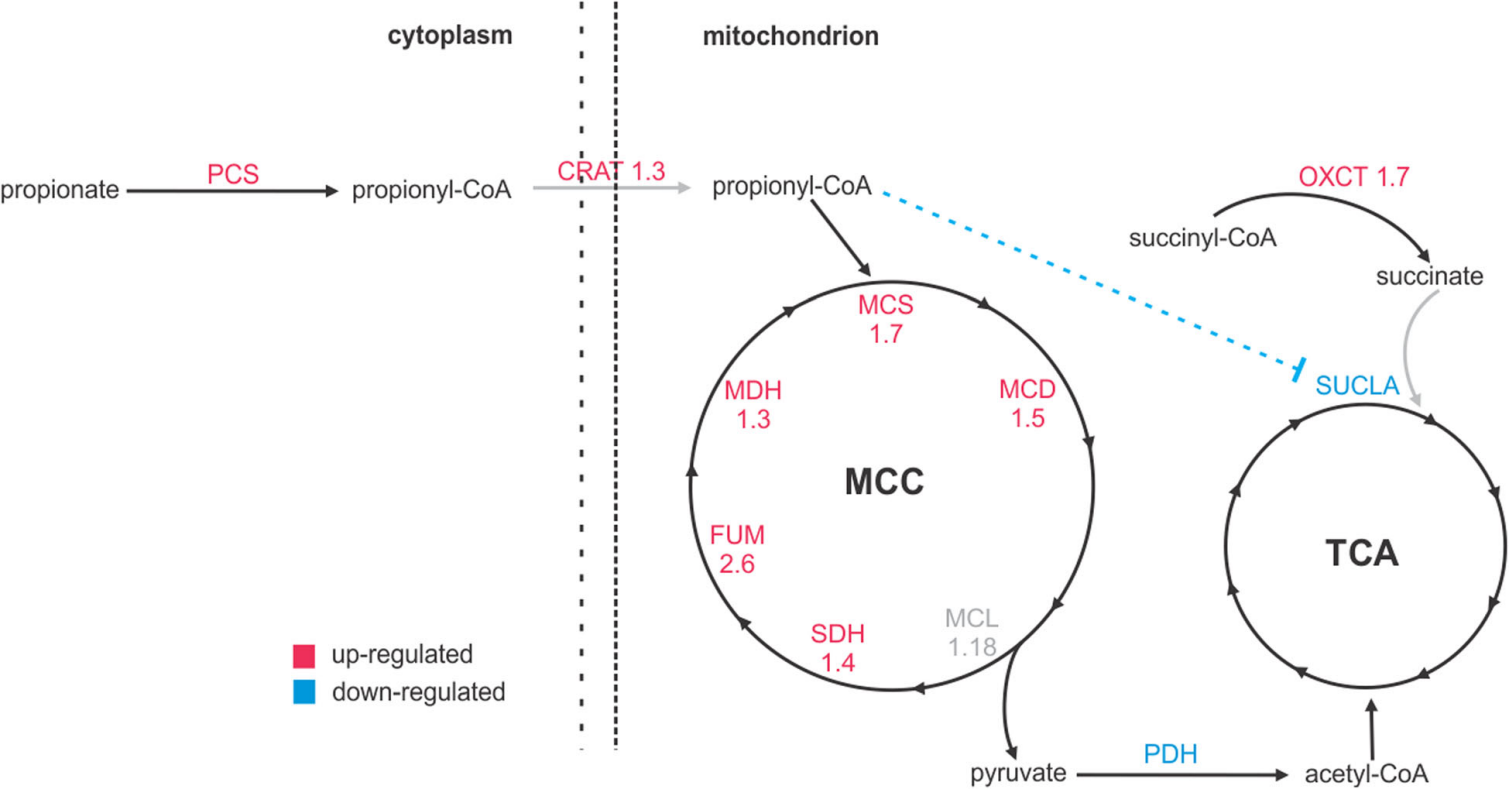
Metabolic processes specifically related to propionyl-CoA turnover. MCS methylcitrate synthase, MCD methylcitrate dehydratase, MCL methylisocitrate lyase, PCS propionyl-CoA-synthase, FUM fumarase, MDH malate dehydrogenase, SDH succinate dehydrogenase, PDH pyruvate dehydrogenase, succinyl-CoA ligase, OXCT succinyl-CoA-3-ketoacid transferase

Our proteomic data also indicate specific mechanisms used by this fungus in propionate consumption (Fig. 7). The methylcitrate cycle specific enzymes MCS and MCD presented an increase of 1.7 and 1.5 fold in response to propionate. Although MCL was not identified as an induced protein using the 1.2 fold cutoff, this protein presented a 1.18 fold increase. The enzymes shared by TCA and MCC such as succinate dehydrogenase, fumarate hydratase and malate dehydrogenase also accumulate in response to propionate, indicating a global induction of MCC activity. Propionyl-CoA strongly inhibits the succinyl-CoA synthetase, which would lock the subsequent reactions of the citric acid cycle and hampers the utilization of pyruvate from both glucose and propionate (Brock and Buckel 2004). However, *Paracoccidioides* spp. yeast cells can grow in the presence of propionate as carbon source and in combination with glucose. Hence, these fungi hold an alternative reaction that converts succinyl-CoA into succinate. Corroborating with this fact the enzyme succinyl-CoA:acyl-CoA transferase, which can transfer the CoA-moiety from succinyl-CoA to a carboxylic acid (probably propionate) yielding succinate for the TCA cycle, was induced 1.7 fold (Fleck and Brock 2008).

**Fig. 8:**
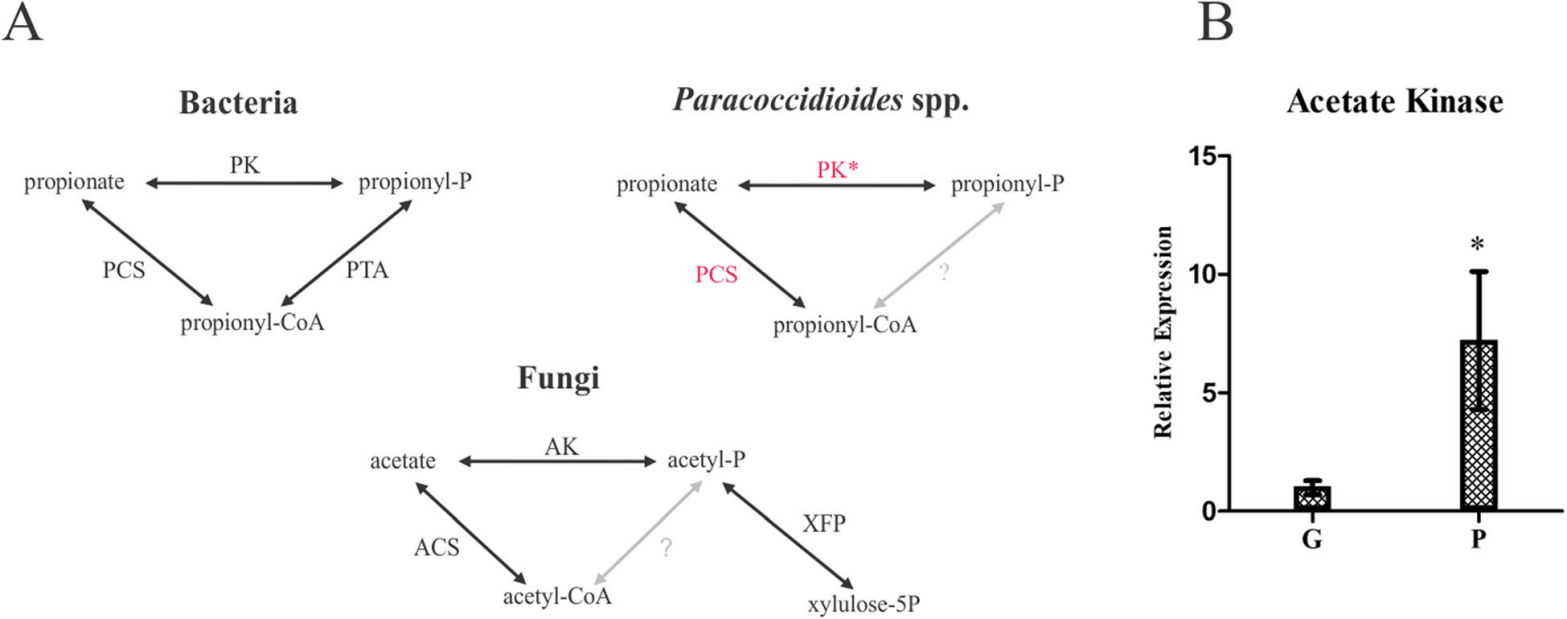
Roles of propionate and acetate kinases in acetate or propionate activation. **a** - AK acetate kinase, PK propionate kinase, PTA phosphotransacetylase, XFP d-xylulose-5-phosphate phosphoketolase, PCS propionyl-CoA syntethase. * Putative propionate kinase found only in propionate condition. **b** - Transcript levels of acetate kinase in yeast cells grown in glucose (G) or propionate (P)

Intriguingly, an acetate kinase (AK) was detected only when the fungus was grown in propionate. This enzyme is described as a component of the acetate activation into acetyl-CoA in a phosphotransacetylase (PTA) dependent fashion. This route is activated in acetate growing bacteria but is not found in fungi since these organisms have no predicted PTA encoding genes (Ingram-Smith et al. 2006). Fungal AK integrates a pathway with d-xylulose 5-phosphate phosphoketolase (XFP), which produces acetate in xylose grown cells (Fig. 8a). Additionally, conversion of propionate into propionyl-CoA in *Neisseria meningitides* relies on enzymes such as propionate kinase (PK) and PTA in an acetate analogous route (Catenazzi et al. 2014). The AK found in our proteomic analysis is homologous to the *N. meningitides* PK, suggesting that the enzyme might act in propionate and propionyl-phosphate interconversion to degrade propionate. PK transcript levels were measured to investigate its putative role in propionate metabolism. The data showed that *Paracoccidioides* sp. presents PK encoding transcripts that accumulate in yeast cells exposed to propionate (Fig. 8b).

## DISCUSSION

The ability to metabolize propionate is an important characteristic of the metabolic flexibility of a microorganism, since it enables the use of propionyl-CoA generating carbon sources in both environmental and host milieus. In the saprobiotic lifestyle, the propionyl-CoA metabolizing organisms may use propionate present in the soil (the second most abundant carbon source in the soil), thus presenting a metabolic advantage over organisms that are unable to metabolize propionate. During infection, microorganisms experience a nutrient poor environment imposed by host defenses and propionyl-CoA turnover capacity enable them to use branched-chain amino acids, odd chain fatty acids and cholesterol as nutrient sources. This broadens the host tissues/niches that the pathogen may colonize and contributes to virulence. Analysis of propionate metabolism in *Paracoccidioides* spp. has shown that MCC enzymes are induced by propionate and, in the case of MCS, the enzyme is regulated by phosphorylation. Several MCS isoforms were detected by two-dimensional immunoblotting and dephosphorylation of protein extracts decreased the enzyme activity by half when compared to untreated controls. Proteomic data suggest that the fungus activates lipid and amino acid degradation to support propionate utilization. Additionally, the proteome approach suggests that a putative propionate kinase may participate in propionate activation and utilization.

The search for genes related to propionate metabolism revealed that *Paracoccidioides* spp. harbor genes coding for methylcitrate cycle enzymes. This characteristic is shared by other pathogenic fungi such as *A. fumigatus* (Maerker et al. 2005), *Fusarium solani* and *Fusarium verticillioides* (Domin et al. 2009) reinforcing the notion that MCC was kept as preferred pathway for use of propionyl-CoA generating sources in fungi. Carbon utilization in filamentous fungi is controlled by a catabolic repressor system. In *A. nidulans,* the carbon catabolite repressor CreA inhibits alternative carbon source metabolic routes when glucose is available. Acting at transcriptional level, CreA represses target genes by binding to consensus sequences (5′-(G/C)YGGRG-3′) at their promoters (Panozzo et al. 1998). A CreA homologue was found in *Paracoccidioides* spp. genomes and the promoter sequences of the three MCC specific genes presented the CreA binding motifs suggesting the MCC is under the carbon catabolite repression.

Although *Paracoccidioides* spp. carry genes putatively encoding for MCC enzymes, to date no experimental evidence has confirmed their functionality. Real time PCR data indicates that MCS, MCD and MCL genes were induced by propionate. Additionally, the MCS protein accumulates in yeast and mycelium cells incubated with propionate and this fact is accompanied by an increase in MCS enzymatic activity. The presence of glucose inhibits MCS activity by a rate of 1.6, which differs from *A. fumigatus*, *F. solani* and *F. verticillioides* that showed inhibition rates between 28 and 7 times (Domin et al. 2009; Ibrahim-Granet et al. 2008). This difference may be explained by the higher MCS activity in glucose grown *Paracoccidioides* cells (0.51 U × mg^− 1^ in yeast and 2.42 U × mg^− 1^ in mycelium) when compared to propionate. Additionally, high MCS activity detected in mycelial cells may reflect an adaptive differential metabolic characteristic of this morphology since, in nature, propionate is the second most abundant carbon source in soil (Buckel 1999). Differences in the metabolic status between *Paracoccidioides* yeast and mycelium phases have been described. Transcriptomic and proteomic approaches suggested that mycelium oxidizes glucose by an aerobic route while yeasts present a more fermentative glucose metabolism in *P. lutzii* (Bastos et al. 2007; Felipe et al. 2005; Rezende et al. 2011). However, proteomic data show the reverse for *P. brasiliensis* (Araujo et al. 2019). Also, enzymes of the methylcitrate and glyoxylate cycles were induced during mycelium to yeast transition (Rezende et al. 2011). Thus, *Paracoccidioides* genus presents a phase specific metabolic characteristic that is not restricted to propionate.

Western blotting analysis demonstrated that mycelium and yeast cells present a different distribution of MCS isoforms. The isoforms have similar molecular masses but different isoelectric point suggesting that MCS is under a phosphorylation-based regulation. To the best of our knowledge this is first description of MCS being regulated by phosphorylation. In *P. lutzii* only one similar event is described for isocitrate lyase (ICL). During growth in acetate, fungal cells promote ICL activation by phosphorylation as a fast mechanism for fungal adaptation (Cruz et al. 2011). Our data suggest that phosphorylation also activates MCS since the activity decreased by at least two fold in dephosphorylated protein extracts. This fact, together with transcript and protein accumulations in propionate treated cells, indicates that MCS is regulated at transcriptional level as well as by post-translational modification. The last regulatory mechanism denotes a new strategy for the rapid adaptation to changing environmental conditions, such carbon sources.

The proteomics approach brings to light the metabolic strategies used by this pathogen to survive in propionate as carbon source. The adaptation to propionate is beyond the activation of propionyl-CoA-detoxifying pathways. Towards energy production *P. lutzii* activates the aerobic metabolism by the accumulation of proteins related to the respiratory chain and decreases the level of the fermentative enzyme alcohol dehydrogenase. Additionally, the fungus operates oxidation of fatty acids and amino acid degradation for ATP production and building blocks for synthesis of essential metabolites. Coping with that, the glyoxylate cycle isocitrate lyase is activated to promote the oxaloacetate anaplerosis into gluconeogenesis, since FBPase1 is also induced. Similar behavior was observed in *Paracoccidioides* sp. yeast cells recovered from mouse lungs, a carbon source poor environment (Lacerda Pigosso et al. 2017), indicating that this response is triggered when preferential sources are not available. This reflects that *P. lutzii* can rewire its metabolism in host niches where alternative carbon sources are available, as shown for other fungal pathogens. Glyoxylate cycle, glycolysis and gluconeogenesis are all required for *C. albicans* virulence in a disseminated mouse infection model (Barelle et al. 2006; Lorenz and Fink 2001). In a paralleled way, *C. neoformans* requires glycolytic (Price et al. 2011) and gluconeogenic (Panepinto et al. 2005) pathways for full virulence. The paradoxical activation of competing pathways may reflect heterogeneous nature of fungal population in complex host microenvironments with different carbon sources (Ene et al. 2014). This metabolic flexibility found in pathogenic fungi is a key aspect of fungal virulence.

Growth in propionate demands activation of specific vitamin biosynthetic routes. Lumazine synthase and riboflavin synthase induction suggests dependence of *P. lutzii* on riboflavin requiring processes (Guenther et al. 1999). Accordingly, fatty acid oxidation and spermidine synthesis related enzymes were also up-regulated by propionate. Fatty acid oxidation relies on FAD dependent enzymes, a derivative compound of riboflavin. Spermidine is derived from SAM (S-adenosylmethionine), a compound also synthetized by FAD-dependent enzymes. This fact is reinforced by the induction of the SAM synthesis proteins methionine synthase, adenosylhomocysteinase and S-adenosylmethionine synthase (Fontecave et al. 2004; Guenther et al. 1999). The increase in spermidine synthesis suggests that this stress related molecule (Valdes-Santiago and Ruiz-Herrera 2013) may be pertinent to survival in the carbon source propionate that produces the toxic intermediate propionyl-CoA. The ability to perform de novo synthesis of vitamins has been reported as essential in host-resembling environments. *A. fumigatus* iron homeostasis and virulence depends on functional riboflavin and pantothenic acid biosynthesis. Riboflavin and pantothenate de novo syntheses are also required for *H. capsulatum* survival in macrophage and for virulence in a murine model of respiratory histoplasmosis (Garfoot et al. 2014). The induction of riboflavin enzymes by *Paracoccidioides* sp. in propionate and the reported link between riboflavin and fungal virulence reinforces the idea that fungi may be exposed to propionyl-CoA generating compounds in host tissues.

Global analysis of proteins levels of yeast cells grown in propionate revealed the strategies used by *P. lutzii* to metabolize and counteract the toxic effects of propionate. As expected, MCS and MCD protein levels accumulated, as well as enzymes of the TCA cycle (succinate dehydrogenase, fumarate hydratase and malate dehydrogenase), which are shared with MCC. The propionyl-CoA metabolism is dependent on a functional TCA cycle; however, succinyl-CoA synthetase is inhibited by propionyl-CoA. Probably the fungus circumvents this blockage by the induction of a succinyl-CoA:acyl-CoA transferase (PAAG_05093) to fuel succinyl-CoA into the TCA cycle (Brock and Buckel 2004; Fleck and Brock 2008). Among the proteins related to propionyl-CoA detoxification, an acetate/propionate kinase was found. In *Neisseria menigitidis* an analogous enzyme participates in the propionyl-CoA/propionyl-phosphate interconversion (Catenazzi et al. 2014) putatively in order to keep the amount of propionyl-CoA at low levels. Thus, we hypothesized that *P. lutzii* may use propionate kinase activity to regulate the levels of propionyl-CoA. The induction of a lactate dehydrogenase (LDH) was surprising since glycolytic enzymes were repressed in propionate. However, lactate dehydrogenase is the final step in the conversion of propionyl-CoA into pyruvate by the acryloyl-CoA pathway. In such process, propionyl-CoA is oxidized in acrylyl-CoA, which in turn is hydrated in lactoyl-CoA. Finally, lactoyl-CoA is cleaved into lactate, which is converted into pyruvate by LDH (Fernandez-Briera and Garrido-Pertierra 1988). Putative enzymes predicted to act as acyl-CoA dehydrogenases and acyl-CoA hydratase were found as induced in our work (PAAG_06329, PAAG_06309, PAAG_04811). However, further efforts need to be done in order to confirm this hypothesis.

## CONCLUSIONS

Our study brought new insights into biochemical and molecular aspects of propionate metabolism in *Paracoccidioides* spp., which may be expanded to other dimorphic fungus. Biochemical data showed, for the first time, that MCS activity is under a phosphorylation regulatory mechanism. Additionally, the global view of fungal metabolism indicates that propionate metabolism requires activation of alternative carbon sources using pathways to support carbon requirement in MCC and TCA. The results also suggest that propionyl-CoA levels may be balanced by a putative propionate kinase in order to buffer the toxicity of this metabolite.

## Supplementary information

**Additional file 1: Table S1.** Primers used in qRT-PCR experiments.

**Additional file 2: Table S2.** Genbank accession numbers of the sequences used in this study.

**Additional file 3: Table S3.** Up-regulated proteins in propionate grown yeast cells.

**Additional file 4: Table S4.** Dow-regulated proteins in propionate grown yeast cells.

**Additional file 5: Figure S1.** Growth analysis of *P. brasiliensis* on propionate. Serial dilutions of cell suspensions (from 10^6^ to 10^3^ cells) were spotted on MMcM containing propionate (Prop), glucose plus propionate (Gluc/Prop) or glucose (Gluc) as carbon sources. The plates were incubated at 36°C for 10 days.

## Data Availability

Not applicable.

## Abbreviation

AK: Acetate kinase
CNS: Central nervous system
CoA: Coenzyme A
FAD: Flavin adenine dinucleotide
FMD: Formamidase
ICL: Isocitrate lyase
LDH: Lactate dehydrogenase
MCC: Methylcitrate cycle
MCD: Methylcitrate dehydratase
MCL: Methylisocitrate lyase
MCS: Methylcitrate synthase
MMcM: McVeigh and Morton medium
PCM: Paracoccidioidomycosis
PCS: Propionyl-CoA synthetase
PK: Propionate kinase
PTA: Phosphotransacetylase
r*Pb*MCS: recombinant *Paracoccidioides* spp. MCS
SAM: S-adenosylmethionine
TCA: Tricarboxylic acid cycle
XFP: D-xylulose 5-phosphate phosphoketolase
