# Supplementary material for: Propionate metabolism in a human pathogenic fungus: proteomic and biochemical analyses
Source: IMA Fungus. 2020 May 5;11:9. doi: 10.1186/s43008-020-00029-9 (PMC7324963; doi:10.1186/s43008-020-00029-9)
Supplement: Supplementary file 1 — Additional file 1: Table S1. Primers used in qRT-PCR experiments. [file 43008_2020_29_MOESM1_ESM.docx]

**Table S1 – Primers used in qRT-PCR experiments.**

| **Primers** | **Sequence** | **Genbank Accession number** |
| --- | --- | --- |
| Tubulin forward | 5’ ACAGTGCTTGGGAACTATACC 3’ | XM_002796593 |
| Tubulin reverse | 5’ GGGACATATTTGCCACTGCC 3’ |  |
| MCS forward | 5’ CATCTCAGTCTGCCGCTTG 3’ | XP_002793640 |
| MCS reverse | 5’ CTCGTTGGCATCCAGGACA 3’ |  |
| MCD forward | 5’ CAACTCTGACCTTGCATTTGAT 3’ | XP_002793649 |
| MCD reverse | 5’ GATGTTGAAAGCACCGTTGAC 3’ |  |
| MCL forward | 5’ CTTGTTACTTTCGATGAAGCGG 3’ | XP_002793639 |
| MCL reverse | 5’ GATGTCCCAGGACCAGAACAC 3’ |  |
| PCS forward | 5’ GATGTTGTCTTGATTTACATGCC 3’ | XP_002789592 |
| PCS reverse | 5’ GTGGTTTCGATGCCTCGATG 3’ |  |
| PK forward | 5’ CTGAAGCAGCCTATCGAGTC 3’ | XP_015700591 |
| PK reverse | 5’ GAGGATGCGTTCAGCCGTG 3’ |  |
