# Supplementary material for: Propionate metabolism in a human pathogenic fungus: proteomic and biochemical analyses
Source: IMA Fungus. 2020 May 5;11:9. doi: 10.1186/s43008-020-00029-9 (PMC7324963; doi:10.1186/s43008-020-00029-9)
Supplement: Supplementary file 2 — Additional file 2: Table S2. Genbank accession numbers of the sequences used in this study. [file 43008_2020_29_MOESM2_ESM.docx]

**Table S2 – Genbank accession numbers of the sequences used in this study**

*Af* – *Aspergillus fumigatus*; *Ce* - *C. elegans*; Ec – *E. Coli*; *Mt - M tuberculosis; Pb*01*,* *Pb*03 e *Pb*18 – *Paracoccidioides* spp. phylogenetic species 01, 03 and 18

| Description | *Af* | *Ce* | *Ec* | *Mt* | *Pb*01 | *Pb*03 | *Pb*18 |
| --- | --- | --- | --- | --- | --- | --- | --- |
| Methylcitrate synthase | XP_747718 | - |  | - | XP_002793640 | EEH22113 | EEH48631 |
| Methylcitrate dehydratase | XP_747704 | - |  | - | XP_002793649 | EEH22120 | EEH48639 |
| Methyliso-citrate lyase | CAW40752 | - |  | - | XP_002793639 | EEH22112 | EEH48630 |
| Propionyl-CoA synthase | - | - | CBF70755 | - | XP_002789592 | EEH22449 | EEH49202 |
| Methylmalonyl-CoA mutase | - | CAA84676 |  | Rv1492 and Rv1493 | - | - | - |
| Propionate-kinase |  |  |  |  | XP_015700591 | EEH20257 | EEH44651 |
