# Supplementary figures and images for: Propionate metabolism in a human pathogenic fungus: proteomic and biochemical analyses
Source: IMA Fungus. 2020 May 5;11:9. doi: 10.1186/s43008-020-00029-9 (PMC7324963; doi:10.1186/s43008-020-00029-9)

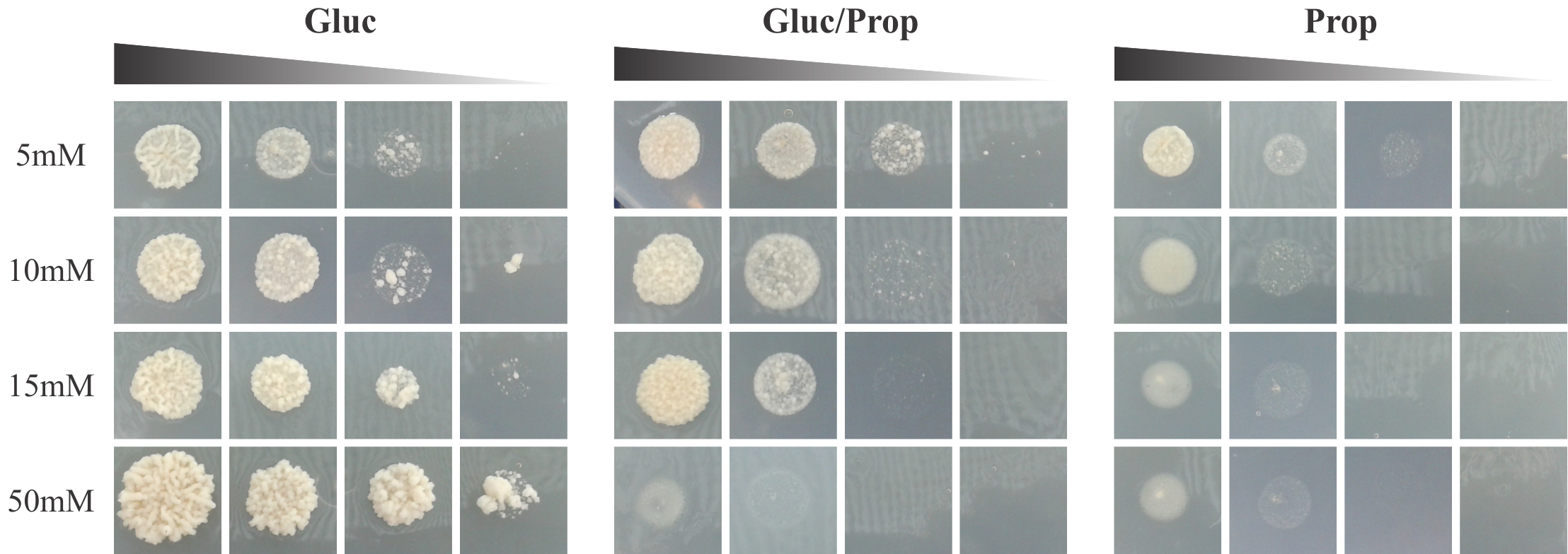

Supplement: Supplementary file 5 — Additional file 5: Figure S1. Growth analysis of P. brasiliensis on propionate. Serial dilutions of cell suspensions (from 106 to 103 cells) were spotted on MMcM containing propionate (Prop), glucose plus propionate (Gluc/Prop) or glucose (Gluc) as carbon sources. The plates were incubated at 36°C for 10 days. [file 43008_2020_29_MOESM5_ESM.tif]
